# Oxygen Generation via Water Splitting by a Novel Biogenic Metal Ion-Binding Compound

**DOI:** 10.1128/AEM.00286-21

**Published:** 2021-06-25

**Authors:** Philip Dershwitz, Nathan L. Bandow, Junwon Yang, Jeremy D. Semrau, Marcus T. McEllistrem, Rafael A. Heinze, Matheus Fonseca, Joshua C. Ledesma, Jacob R. Jennett, Ana M. DiSpirito, Navjot S. Athwal, Mark S. Hargrove, Thomas A. Bobik, Hans Zischka, Alan A. DiSpirito

**Affiliations:** a Roy J. Carver Department of Biochemistry, Biophysics and Molecular Biology, Iowa State University, Ames, Iowa, USA; b Department of Civil and Environmental Engineering, University of Michigan, Ann Arbor, Michigan, USA; c Materials Science and Biomedical Engineering, University of Wisconsin—Eau Claire, Eau Claire, Wisconsin, USA; d Institute of Molecular Toxicology and Pharmacology, Helmholtz Center Munich, German Research Center for Environmental Health, Neuherberg, Germany; e Technical University Munich, School of Medicine, Institute of Toxicology and Environmental Hygiene, Munich, Germany; University of California, Davis

**Keywords:** methanobactin, chalkophore, water oxidation, methanotroph, aerobic methane oxidation, gold nanoparticle

## Abstract

Methanobactins (MBs) are small (<1,300-Da) posttranslationally modified copper-binding peptides and represent the extracellular component of a copper acquisition system in some methanotrophs. Interestingly, MBs can bind a range of metal ions, with some being reduced after binding, e.g., Cu^2+^ reduced to Cu^+^. Other metal ions, however, are bound but not reduced, e.g., K^+^. The source of electrons for selective metal ion reduction has been speculated to be water but never empirically shown. Here, using H_2_^18^O, we show that when MBs from *Methylocystis* sp. strain SB2 (MB-SB2) and Methylosinus trichosporium OB3b (MB-OB3) were incubated in the presence of either Au^3+^, Cu^2^, or Ag^+^, ^18,18^O_2_ and free protons were released. No ^18,18^O_2_ production was observed in the presence of either MB-SB2 or MB-OB3b alone, gold alone, copper alone, or silver alone or when K^+^ or Mo^2+^ was incubated with MB-SB2. In contrast to MB-OB3b, MB-SB2 binds Fe^3+^ with an N_2_S_2_ coordination and will also reduce Fe^3+^ to Fe^2+^. Iron reduction was also found to be coupled to the oxidation of 2H_2_O and the generation of O_2_. MB-SB2 will also couple Hg^2+^, Ni^2+^, and Co^2+^ reduction to the oxidation of 2H_2_O and the generation of O_2_, but MB-OB3b will not, ostensibly as MB-OB3b binds but does not reduce these metal ions. To determine if the O_2_ generated during metal ion reduction by MB could be coupled to methane oxidation, ^13^CH_4_ oxidation by Methylosinus trichosporium OB3b was monitored under anoxic conditions. The results demonstrate that O_2_ generation from metal ion reduction by MB-OB3b can support methane oxidation.

**IMPORTANCE** The discovery that MB will couple the oxidation of H_2_O to metal ion reduction and the release of O_2_ suggests that methanotrophs expressing MB may be able to maintain their activity under hypoxic/anoxic conditions through the “self-generation” of dioxygen required for the initial oxidation of methane to methanol. Such an ability may be an important factor in enabling methanotrophs to not only colonize the oxic-anoxic interface where methane concentrations are highest but also tolerate significant temporal fluctuations of this interface. Given that genomic surveys often show evidence of aerobic methanotrophs within anoxic zones, the ability to express MB (and thereby generate dioxygen) may be an important parameter in facilitating their ability to remove methane, a potent greenhouse gas, before it enters the atmosphere.

## INTRODUCTION

Aerobic methane-oxidizing bacteria (methanotrophs) oxidize methane to carbon dioxide via a series of two-electron steps with methanol, formaldehyde, and formate as intermediates (1). The initial oxidation of methane to methanol is an oxygen- and energy-dependent reaction and is catalyzed by either a soluble cytoplasmic methane monooxygenase (sMMO) or a particulate or membrane-associated methane monooxygenase (pMMO) (1–8). The reductant for the initial oxidation of methane is supplied by NADH for the sMMO and by quinols for the pMMO (2, 3, 9–11). Methanol is oxidized to formaldehyde by a calcium- or rare-earth-dependent methanol dehydrogenase using a *c*-type cytochrome as an electron acceptor (12–17). Formaldehyde is either assimilated or oxidized using either NAD^+^ or quinone as the electron acceptor (10, 18–20). The final two-electron oxidation of formate to carbon dioxide is catalyzed by the NAD^+^-linked formate dehydrogenase (21–23). Electrons from NADH, quinol, or cytochrome *c* are either utilized in biosynthetic reactions or oxidized for energy using either dioxygen (11), nitrate (24), or ferric iron (25) as the terminal electron acceptor.

In methanotrophs capable of expressing both forms of the MMO, expression is regulated by copper (1, 9, 26–28). In addition to the MMOs, a number of genes are regulated by copper (1), and some methanotrophs of the *Alphaproteobacteria* have novel copper acquisition systems based on the extracellular copper-binding peptide methanobactin (MB) (29–31). MBs are low-molecular-mass (<1,300-Da), high-potential (*E_m_* of 483 to 745 mV) ribosomally synthesized posttranslationally modified peptides (RiPPs) and were the first examples of a chalkophore, i.e., a compound excreted by bacteria for the purpose of scavenging copper from the surrounding environment (30, 32). Structurally, MBs are divided into two groups. Both group I and II MBs are characterized by an internal oxazolone group with an associated thioamide and a second N-terminal 5- or 6-membered ring, which in group I MBs is either an oxazolone or a pyrazinedione group with an associated thioamide, while group II MBs have either an imidazoline or a pyrazinedione group with an associated thioamide (30, 32–36). The ring and associated thioamide are derived from an X-Cys dipeptide via a series of partially characterized posttranslational modifications (29, 31, 37). Group I MBs are characterized by an internal disulfide bridge and the copper-bound form of a dicyclic structure (32–34). Group II MBs lack this disulfide bridge, and the copper-bound form has a hairpin-like structure and is characterized by a central sulfonated threonine (30, 36).

In addition to copper ions, MBs will bind many metal ions (38–41) and reduce some but not all metal ions that are bound (38, 42). In MB from Methylosinus trichosporium OB3b (MB-OB3b), metal ions such as copper, silver, and gold are coordinated via an N_2_S_2_ ligand set utilizing an N from each ring and the two thioamides, and these metals are reduced after binding (30, 34, 38, 43). Other metal ions such as iron, nickel, and cobalt are coordinated via an N_1_S_1_ ligand set using one ring and its associated thioamide and are not reduced (38). Based on coordination, metals were classified as either group A metals coordinated by an N_2_S_2_ ligand set or group B metals coordinated by an N_1_S_1_ ligand set. In contrast, all of the metals bound by MB from *Methylocystis* sp. strain SB2 (MB-SB2) are coordinated by an N_2_S_2_ ligand set (39, 40, 42, 44; this study).

Since metal ion reduction assays are often carried out in unbuffered reaction mixtures in the absence of an external reductant, water has been proposed, but not shown, to be the electron donor (36). Here, we examine the binding and reduction of oxidized forms of gold (as HAuCl_4_), copper (as CuCl_2_), silver (as AgF), iron (as FeCl_3_), nickel (as NiCl_2_), mercury (HgCl_2_), cobalt (as CoCl_2_), and potassium (as KCl) in the presence and absence of H_2_^18^O by MB-SB2 as well as the binding and reduction of gold, copper, and silver in the presence of H_2_^18^O by MB-OB3b.

## RESULTS

### Spectral and thermodynamic properties of AuCl_4_^−^ binding by MB-SB2.

UV-visible absorption, fluorescence, and circular dichroism spectra (see Fig. S1 to S3 in the supplemental material) and thermodynamic measurements (Fig. S4 and Table S1) demonstrate that changes following the addition of HAuCl_4_ to MB-SB2 were complex, with transitions being apparent at 0.25, 0.5, 0.75, 1.0, and 2.0 Au per MB-SB2. As MB-SB2 has only one identified metal-binding motif (i.e., an N_2_S_2_ ligand set), we therefore interpret these data to indicate changes in Au coordination, when MB-SB2 transitions from an oligomeric state(s) to a monomer.

The increased fluorescence emission intensity following HAuCl_4_ addition may be due to disruption of internal quenching between the imidazolone and oxazolone groups and is consistent with the intramolecular exciton transfer previously demonstrated following hydrolysis of the oxazolone group (42) (Fig. S2). The decreased fluorescence at HAuCl_4_/MB-SB2 ratios of >1.0 suggests direct metal quenching or intra-/interexciton transfer between the oxazolone and imidazolone groups or may be associated with nanoparticle formation, which occurs at Au/MB-SB2 ratios of >1:1.

As Au nanoparticle formation requires Au^3+^ reduction (45), nanoparticle formation (Fig. S5 and Table S2) by MB-SB2 indicated that MB-SB2 binds and reduces multiple Au^3+^ molecules to Au^0^. Such findings accentuated the need to determine the electron source for metal reduction by MBs. To extend these preliminary studies, we examined the reduction of HAuCl_4_ via MB-SB2 when dissolved in either H_2_^16^O or H_2_^18^O.

### X-ray photoelectric spectroscopy, kinetics, and chloride determination.

**FIG 1:**
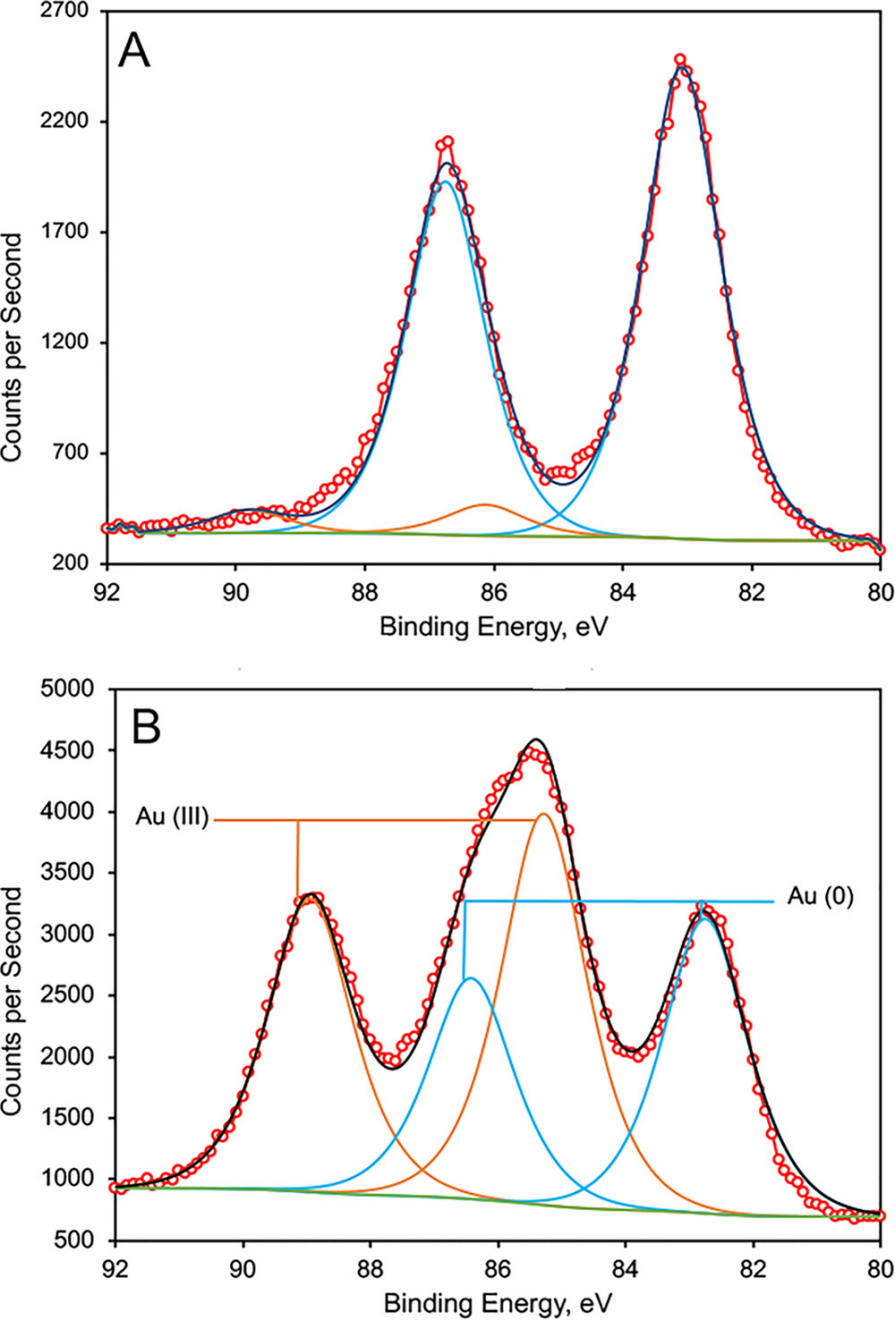
Gold X-ray photoelectric spectra of MS-SB2 at a gold/MB-SB2 molar ratio of 14 to 1 after a 30-min incubation (red circles) (A) and at a gold/MB-SB2 molar ratio of 19 to 1 after a 30-min incubation (B). Experimental results (circles) were fit with CASA XPS software to four Gaussian/Lorentzian curves, using two peaks for Au^3+^ (orange curves) and two peaks for Au^0^ (blue curves). Gold 4f core electrons are spin-orbit split as 4f_7/2_ and 4f_5/2_, with a splitting of 3.7 eV and area ratio of 4:3, so that only two peaks are independently fit: the 4f_7/2_ peaks for Au^3+^ and Au^0^. The 4f_5/2_ peaks’ positions and areas are determined by spin-orbit splitting; these parameters and the peak widths are fixed in the fitting program. The background used was of a Shirley type.

In reaction mixtures containing HAuCl_4_ and MB-SB2 dissolved in H_2_^16^O, MB-SB2 was observed to reduce AuCl_4_^−^ to Au^0^ plus 4Cl^−^, as determined by X-ray photoelectric spectroscopy (XPS) and argentometric titrations, respectively (Fig. 1 and Table 1). MB-SB2 reduced up to 19 Au^3+^ to 19 Au^0^ with a time-dependent average Au^3+^-to-Au^0^ reduction rate of 0.3 ± 0.06 min^−1^ for those assays where rates could be determined. This time-dependent reduction was the reason why samples were frozen in liquid nitrogen and lyophilized overnight to stop the reaction and dry the samples for analysis. As observed with MB-OB3b (38), Au^3+^ and Au^0^ were the only oxidation states detected, indicating a direct three-electron reduction of HAuCl_4_ (Fig. 1).

**TABLE 1:** Distribution of Au as Au^3+^ and Au^0^ following incubation of MB-SB2 and HAuCl_4_^*a*^

HAuCl_4_/MB-SB2 ratio	Time (min)	% Au^3+^	% Au^0^	Reduction rate (Au^3+^ reduced min^−1^)
0.9	30	0	100	
2.25	30	0	100	
9	30	8	92	0.27
14	30	11	89	0.41
19	30	59	41	0.26
9	60	0	100	
14	60	0	100	
19	60	10	90	0.28
19	360	0	100	

a Reduction rates were determined from samples where <100% reduction was observed.

### Kinetics of AuCl_4_^−^ binding and reduction.

**FIG 2:**
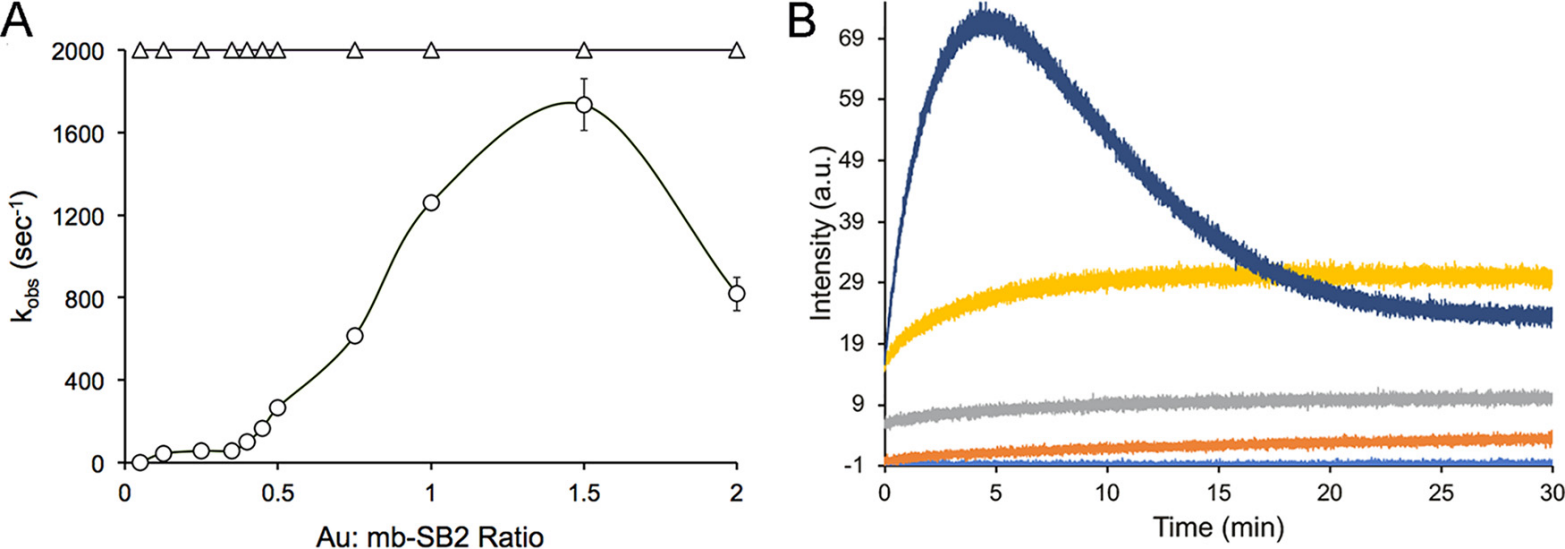
(A) Kinetics of Au binding by MB-SB2 at 4°C. (A) Rate of HAuCl_4_ binding to the imidazolone (△) and oxazolone (○) rings of MB-SB2 at 4°C as measured from the absorbance changes at 386 nm and 341 nm, respectively. The rates for Au binding were >2,000 s^−1^ and were set at 2,000 s^−1^ in the figure. (B) Emission at 429 nm from SB2-MB following excitation at 341 nm after the addition of 0 (light blue), 0.25 (orange), 0.5 (gray), 0.75 (yellow), or 2.25 (dark blue) HAuCl_4_ per MB-SB2. a.u., arbitrary units.

The time course for the binding of Au^3+^ to the oxazolone and imidazolone rings in MB-SB2 was measured as the decrease in the absorbances at 341 and 389 nm, respectively, following stopped-flow mixing of MB-SB2 with Au^3+^ at 4°C (Fig. 2A). Unfortunately, even at 4°C, initial binding rates could be determined only for the oxazolone ring since binding to the imidazolone ring was complete before the mixing of the sample was complete (1.4 ms). In contrast, the rates of binding to the oxazolone ring were low, 12 to 57 s^−1^, at Au^3+^/MB-SB2 ratios of <0.3 and increased at Au^3+^/MB-SB2 ratios of between 0.3 and 1.5 Au^3+^ per MB-SB2, up to a maximum rate of ∼1,600 s^−1^, followed by a decline in the rate at molar ratios of >1.5 Au^3+^ per MB-SB2 (Fig. 2A).

Au^3+^ reduction rates (0.3 ± 0.06 min^−1^) (Table 1) were much lower than the initial binding rates (>2,000 s^−1^) (Fig. 2A). The difference may be due to the different rates of binding between the imidazolone and oxazolone groups. Monitoring the fluorescence changes over time at HAuCl_4_/MB-SB2 ratios of below 1:1 suggested that final Au coordination required several minutes to complete (Fig. 2B). At gold/MB-SB2 ratios of >1.0, an initial disruption of exciton coupling resulted in an increased fluorescence intensity followed by quenching (Fig. 2B). What is pertinent to this discussion is that Au initially binds primarily if not exclusively to the imidazolone group, followed by binding to the oxazolone group and a final reorientation. The time scales for these changes are in keeping with gold reduction rates.

### Oxidation of H_2_O coupled to Au^3+^ reduction by MB-SB2.

**FIG 3:**
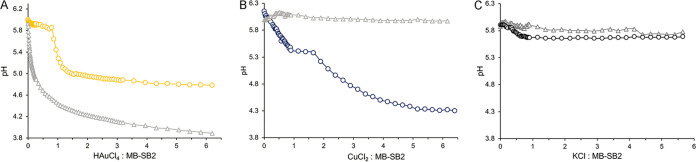
(A) pH changes following the addition of HAuCl_4_ to aqueous solutions (gray triangles) or an aqueous solution of 40 μM MB-SB2 (yellow circles). (B) pH changes following the addition of CuCl_2_ to aqueous solutions (gray triangles) or an aqueous solution of 40 μM MB-SB2 (blue circles). (C) pH changes following the addition of KCl to aqueous solutions (gray triangles) or an aqueous solution of 40 μM MB-SB2 (black circles).

**FIG 4:**
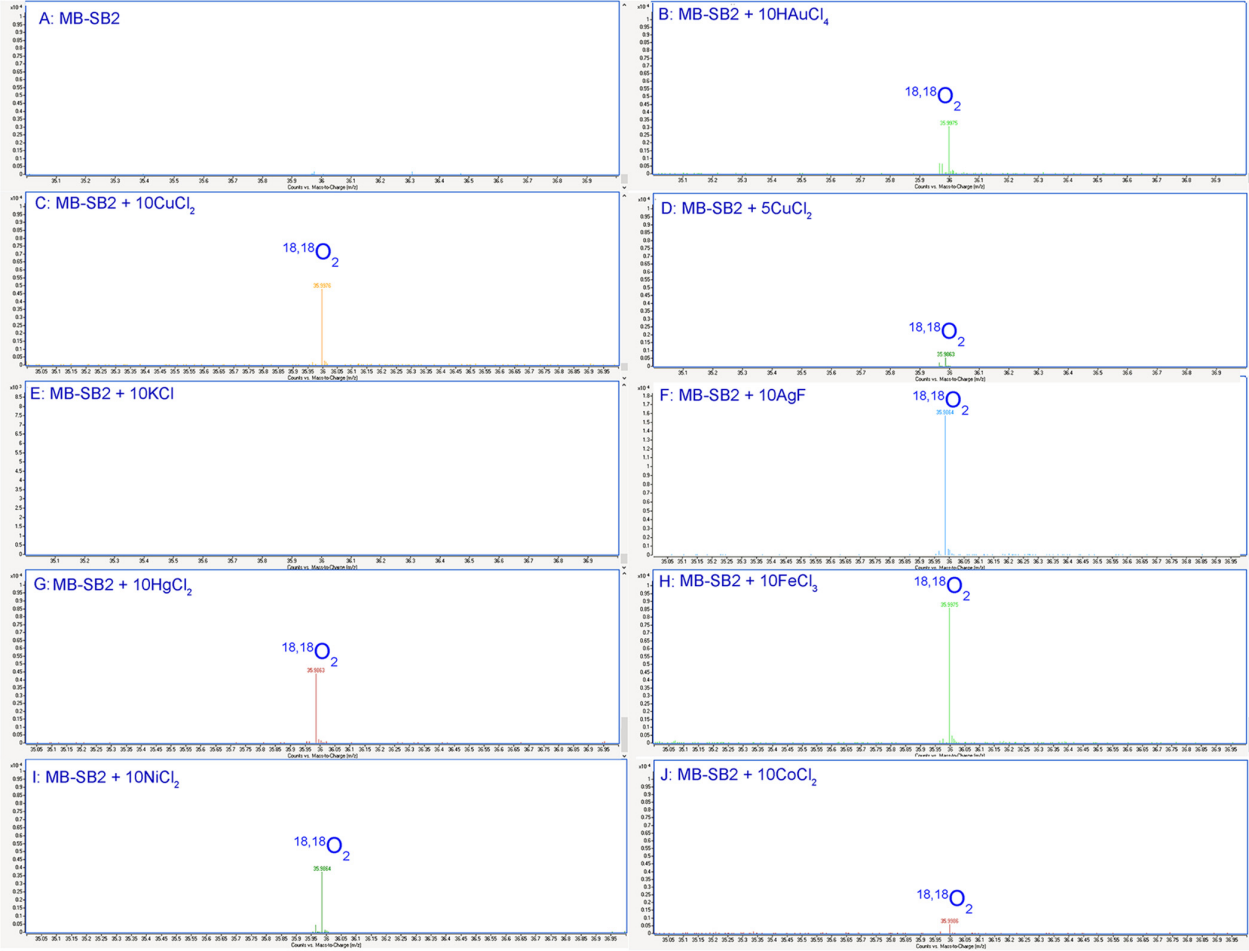
Mass spectra of the headspace gas of a reaction mixture containing 2 mM MB-SB2 in 97% H_2_^18^O (A) and following the addition of 20 mM HAuCl_4_ (B), 20 mM CuCl_2_ (C), 10 mM CuCl_2_ (D), 20 mM KCl (E), 20 mM AgF (F), 20 mM FeCl_3_ (G), 20 mM HgCl_2_ (H), 20 mM NiCl_2_ (I), and 20 mM CoCl_2_ (J).

**FIG 5:**
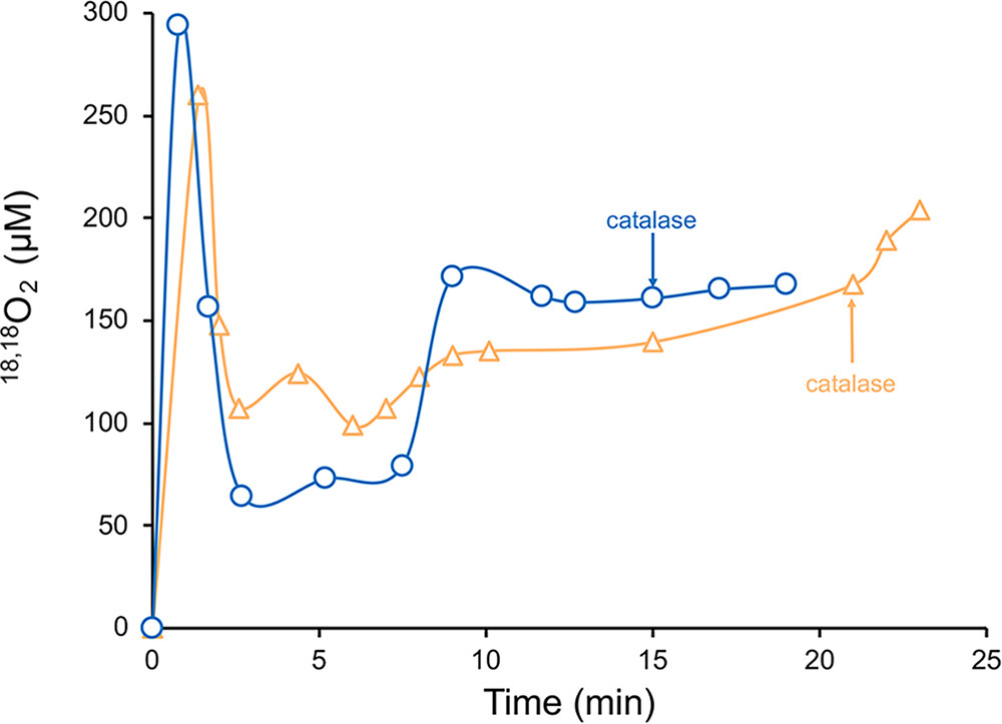
^18,18^O_2_ concentration in the headspace of a reaction mixture containing 2 mM MB-SB2 plus 20 mM HAuCl_4_ (yellow triangles) or 20 mM CuCl_2_ (light blue circles) in 97% H_2_^18^O and following the addition of 7.3 mM catalase.

As four Cl^−^ molecules were generated in reaction mixtures for every HAuCl_4_ molecule reduced to Au^0^, chlorine was ruled out as a potential electron donor. To determine if H_2_O was the electron donor, H^+^ concentrations were monitored during HAuCl_4_ titrations of MB-SB2. Unfortunately, pH changes associated with the addition of HAuCl_4_ to unbuffered reaction mixtures made pH changes associated with the binding of AuCl_4_^−^ difficult to determine (Fig. 3A). To examine if H_2_O could serve as an electron source for Au^3+^ reduction, ^18,18^O_2_ production was monitored in reaction mixtures containing 97% H_2_^18^O. No ^18,18^O_2_ production was observed in reaction mixtures containing either MB-SB2 alone (Fig. 4A) or HAuCl_4_ alone (results not shown). However, following HAuCl_4_ addition to MB-SB2, ^18,18^O_2_ was observed, demonstrating the coupling of water oxidation with metal reduction (Fig. 4B and Fig. 5).

It should be noted that there is seemingly an electron imbalance, with three electrons being required for Au^3+^ to Au^0^, while four electrons are released for every two molecules of water oxidized. There are two possibilities to resolve this issue (46): (i) the reduction of four atoms of Au^3+^ is coupled to the oxidation of six molecules of water, and (ii) the reduction of one atom of Au^3+^ is coupled to the oxidation of two molecules of water, with the fourth electron being used to reduce dioxygen to superoxide. Assays show that reduced MB-SB2 will reduce dioxygen to superoxide (Table S3). Under the low-pH conditions following HAuCl_4_ addition to MB-SB2 (Fig. 3), superoxide would be expected to undergo dismutation reactions, generating H_2_O_2_ (47). In addition, as observed for MB-OB3b (48), Au–MB-SB2 complexes show superoxide dismutase activity (Table S3). Thus, H_2_O_2_ should appear in reaction mixtures if the fourth electron was used to reduce dioxygen. The rate of ^18,18^O_2_ production increased by approximately 18% following the addition of catalase, suggesting the production of H_2_O_2_ (Fig. 5).

### Oxidation of H_2_O coupled to Cu^2+^ reduction by MB-SB2.

Although the oxidation of water to dioxygen coupled to Au^3+^ reduction is chemically interesting, it is not likely biologically relevant. To determine if the oxidation of H_2_O was specific to Au^3+^ reduction or a more general property of metal ion reduction by MB-SB2, similar experiments in H_2_^18^O were carried out with CuCl_2_ as it is believed that the primary purpose of MB is the collection of copper critical for methanotrophic activity. Previous spectral and thermodynamic studies have shown that MB-SB2 will reduce multiple Cu^2+^ molecules to Cu^+^ in the absence of an external reductant, suggesting that water served as the reductant (42). ^18,18^O_2_ evolution was observed following the addition of CuCl_2_ to an H_2_^18^O solution of MB-SB2 (Fig. 4C). Furthermore, such evolution followed a trend similar to that for HAuCl_4_, and a substantial pH drop was observed (Fig. 3B to Fig. 5). Perhaps of greater environmental relevance is the finding of substantial (>100 μM) evolution of dioxygen from water oxidation when MB-SB2 bound and reduced copper (Fig. 5).

The ratio of AuCl_4_^−^ and Cu^2+^ to MB-SB2 in the experiments described above, as well as other metals showing ^18,18^O_2_ production (Fig. 4) described below, was 10:1. To determine the number of electrons needed to be extracted from MB-SB2 before water oxidation occurs, reaction mixtures containing 0.5, 1, 2, 3, 4, and 5 Cu^2+^ per MB-SB2 in 97% H_2_^18^O were examined. No ^18,18^O_2_ was observed in samples containing 0.5, 1, 2, 3, or 4 Cu^2+^ per MB-SB2 (results not shown). ^18,18^O_2_ was observed in samples containing 5 Cu^2+^ per MB-SB2, indicating that for the initial water oxidation to occur, five electrons must be extracted from MB-SB2 (Fig. 4D).

K^+^ was also examined as a metal ion bound by MB-SB2 (Fig. S6A) but not reduced, as no evidence of the formation of metallic K^0^ was observed (49). No ^18,18^O_2_ was observed following the addition of KCl (Fig. 4E), and comparatively minor changes in pH (Fig. 3C) were observed, demonstrating that water oxidation by MB-SB2 after binding a metal ion is contingent upon that metal being reduced. MB-SB2 does not bind Mo^2+^ (Fig. S6B) and was used as a negative control. As expected, no ^18,18^O_2_ was observed in reaction mixtures containing NaMoO_4_ and MB-SB2 (results not shown).

### Oxidation of H_2_O coupled to Ag^+^, Hg^2+^, Fe^3^, Ni^2+^, and Co^2+^ reduction by MB-SB2.

As described above, group A metal ions bound by MB-OB3b are reduced following binding. Ag^+^ and Hg^2+^ are group A metals; MB-SB2 bound both metals via an N_2_S_2_ coordination (Fig. S6C and D), and ^18,18^O_2_ was observed in reaction mixtures containing MB-SB2 and AgF (Fig. 4F) or HgCl_2_ (Fig. 4G) at levels similar to those observed with gold and copper.

**FIG 6:**
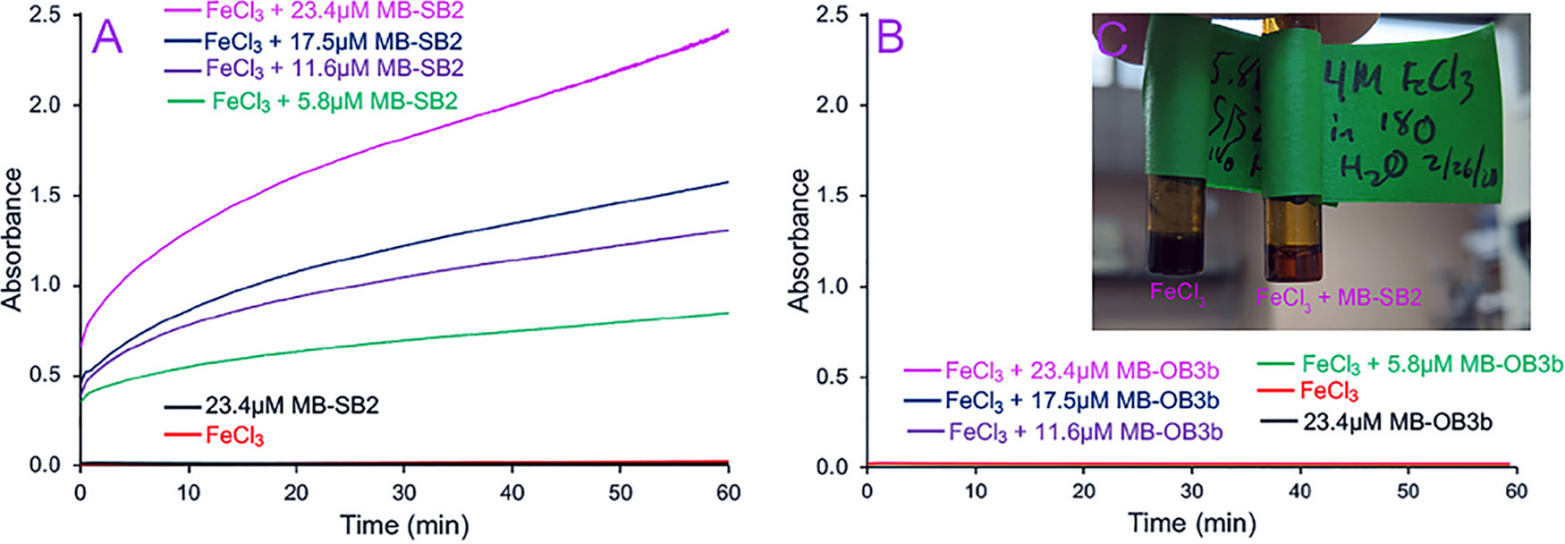
(A and B) Iron reductase activities of MB-SB2 (A) and MB-OB3b (B). The absorption change at 562 nm of reaction mixtures containing 1 mM ferrozine plus 10 mM FeCl_3_, 1 mM ferrozine plus 23.4 μM MB-SB2, 1 mM ferrozine plus 10 mM FeCl_3_, and either 5.8, 11.6, 17.4, or 23.4 μM MB-SB2 (A) or MB-OB3b (B) was measured. (C) Aqueous 4 M FeCl_3_ solution and 4 M FeCl_3_ solution plus 20 mM MB-SB2 4 h after the addition of MB-SB2.

In contrast to MB-OB3b (38), MB-SB2 binds all metal ions tested via an N_2_S_2_ coordination (39, 40, 42) (Fig. S6). Also, in contrast to MB-OB3b, MB-SB2 will reduce Fe^3+^ to Fe^2+^ at a rate of 1.02 ± 0.09 min^−1^ as measured via the ferrozine assay (50, 51) (Fig. 6A). In fact, MB-SB2 will dissolve insoluble Fe^3+^ hydroxides in the light (Fig. S6E, inset) or dark (Fig. 6C). The one-electron ferric iron reduction rate was approximately three times higher than the three-electron gold reduction rate. In reaction mixtures containing MB-SB2 and FeCl_3_ (Fig. 4H), ^18,18^O_2_ was observed at concentrations 1.3- ± 0.1-fold higher than those observed with Au^3+^ and Cu^2+^. ^18,18^O_2_ was also observed in reaction mixtures containing NiCl_2_ (Fig. 4I and Fig. S6F) or CoCl_2_ (Fig. 4H and Fig. S6G) and MB-SB2, although the concentration of ^18,18^O_2_ was consistently low with CoCl_2_.

### Oxidation of H_2_O coupled to Au^3+^, Cu^2+^, and Ag^+^ reduction by MB-OB3b.

**FIG 7:**
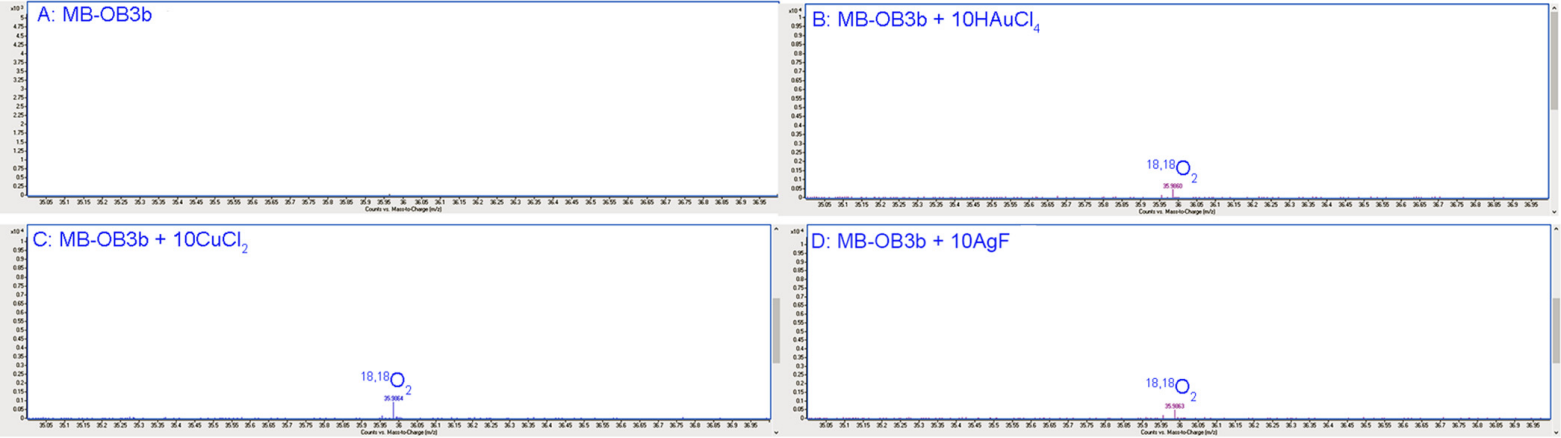
Mass spectra of the headspace gas of a reaction mixture containing 2 mM MB-OB3b in 97% H_2_^18^O (A) and following the addition of 20 mM HAuCl_4_ (B), 20 mM CuCl_2_ (C), and 20 mM AgF (D).

To determine if water oxidation coupled to metal ion reduction was specific to MB-SB2, a group II MB, or a more general property of MBs, water oxidation was examined in the group I MB from M. trichosporium OB3b (MB-OB3b) (31). Previous studies have shown that MB-OB3b binds and reduces Au^3+^, Cu^2+^, and Ag^+^ to Au^0^, Cu^+^, and Ag^0^, respectively, and binds but does not reduce Fe^3+^ (Fig. 6B) (38, 43). Thus, ^18,18^O_2_ production was monitored in reaction mixtures containing HAuCl_4_, CuCl_2_, AgF, and FeCl_3_ with or without MB-OB3b prepared in 97% H_2_^18^O. Again, no ^18,18^O_2_ production was observed in reaction mixtures containing MB-OB3b either alone (Fig. 7A) or with HAuCl_4_, CuCl_2_, or AgF alone (results not shown). However, following HAuCl_4_ (Fig. 7B), CuCl_2_ (Fig. 7C), or AgF (Fig. 7D) addition to reaction mixtures containing MB-OB3b, ^18,18^O_2_ was observed, although the concentrations of ^18,18^O_2_ were <25% of the ^18,18^O_2_ concentrations produced in similar reactions with MB-SB2. No ^18,18^O_2_ was observed following FeCl_3_ addition to a reaction mixture containing MB-OB3b.

### Methane oxidation coupled to O_2_ generated from Cu^2+^ reduction by MB-OB3b.

**FIG 8:**
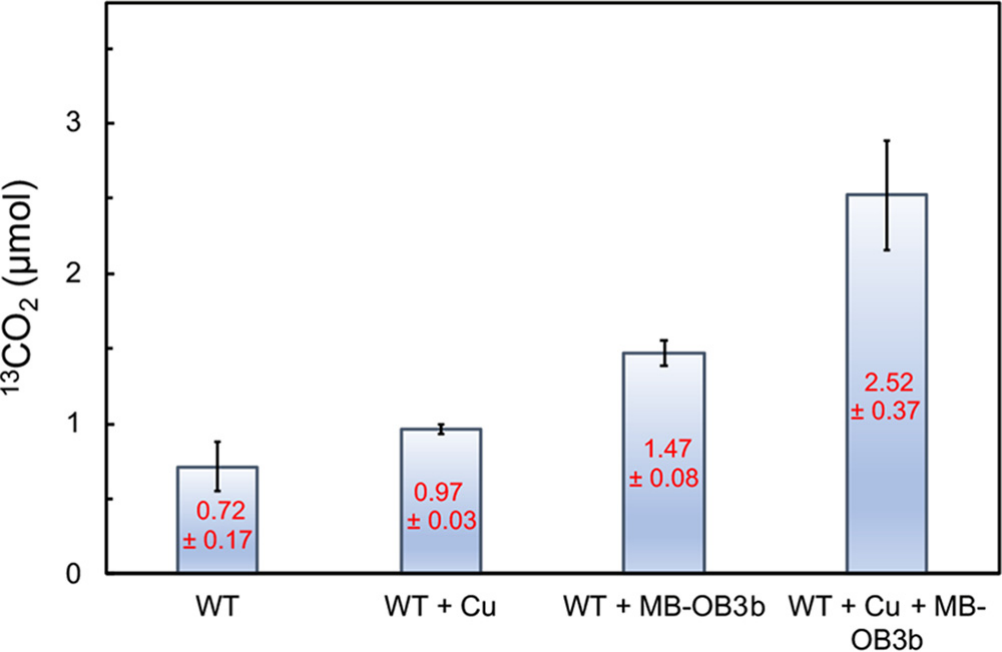
^13^CO_2_ production by the *M. trichosporium* OB3b wild type (WT), the WT plus 25 μM CuCl_2_, the WT plus 5 μM MB-OB3b, and the WT plus 25 μM CuCl_2_ and 5 μM MB-OB3b incubated in an anaerobic glove box for 3 days.

To determine if dioxygen generated during metal ion reduction could support methane oxidation by *M. trichosporium* OB3b, incubations with ^13^CH_4_ in the presence and absence of MB-OB3b and Cu^2+^ were performed under anoxic conditions in an anaerobic glove box. In cell suspensions with no additional amendments of either copper or MB-OB3b, 0.72 ± 0.17 μmol ^13^CO_2_ was observed after 3 days (assumed to be driven by the presence of residual dioxygen in the reaction mixtures [Fig. 8]). In cell suspensions amended with 25 μM Cu^2+^, 0.97 ± 0.03 μmol ^13^CO_2_ was observed (a 34% increase, not significantly different from the amount of ^13^CO_2_ measured with no amendment [*P* = 0.06]). If 5 μM MB-OB3b was added instead, 1.47 ± 0.08 μmol ^13^CO_2_ was measured (an increase of ∼104%, significantly higher than with no amendment [*P* = 2.2 × 10^−3^], presumably due to MB-OB3b binding and reducing metals that are part of the growth medium). If both 25 μM Cu and 5 μM MB-OB3b were added, 2.5 ± 0.37 μmol ^13^CO_2_ was observed, an increase of ∼250% from that with no amendment (again, significantly different [*P* = 1.5 × 10^−3^]), indicating that metal ion reduction by MB can support methane oxidation under anoxic conditions (Fig. 8).

## DISCUSSION

Metal ion binding by MBs has focused on MB-OB3b, a group I MB (34, 38, 43). MB-OB3b bound group A metal ions such as Cu^2+^, Au^3+^, Ag^+^, and Hg^2+^ via an N_2_S_2_ ligand set. Other metal ions such as Fe^3+^, Co^2+^, Cd^2+^, Mn^2+^, Ni^2+^, and Zn^2+^ showed an N_1_S_1_ coordination and were placed in group B. Of the metal ions examined, group A metals were reduced following binding, whereas group B metal ions were not. In this and previous reports (39, 40, 42, 44), MB-SB2, a group II MB, coordinates all metals bound via N_2_S_2_ coordination and reduced metals previously placed in groups A and B. With the exception of K^+^, metal ions bound via an N_2_S_2_ coordination are reduced, and here, we show that H_2_O can serve as an electron donor driving metal ion reduction.

The finding that MB, after binding specific metal ions, can split water to form dioxygen and that this reaction can drive methane oxidation under anoxic conditions suggests that this may be another strategy whereby aerobic methanotrophy can occur in an anoxic environment. That is, it has been shown that methane oxidation via aerobic methanotrophy occurs in anoxic zones of shallow lakes (i.e., at a depth of ∼10 m), with such activity being driven by oxygenic photosynthesis as sunlight could penetrate to this depth (52, 53). In these studies, it was found that methane oxidation rates increased in the light versus the dark, and such activity was abolished when a selective inhibitor of photosynthesis was added. Thus, it appears that methanotrophs can form very effective relationships with oxygenic photosynthetic microbes to scavenge trace amounts of dioxygen and, by so doing, enhance methane removal from these environments.

More germane to the findings here, however, is the discovery that aerobic methanotrophs were also active in deep lake water (∼160 m) where oxygenic photosynthesis is highly unlikely as sunlight cannot penetrate to this depth (54). Such activity, however, could be stimulated by the addition of dioxygen and oxidized metals. Here, it was concluded that methanotrophs may survive anoxic environments by utilizing alternative electron acceptors. Others have shown that aerobic methanotrophs of the *Methylobacter* genus can be stimulated in anoxic lake waters through the addition of either nitrate or sulfate (55). Indeed, it has been shown that some aerobic methanotrophs can respire nitrate (24) or ferric iron (25). Such a strategy could conserve trace amounts of dioxygen to enable methane oxidation by the MMOs. Alternatively, it has been shown that some methanotrophs will couple methane oxidation to fermentation to putatively conserve dioxygen (56), and such a strategy has been speculated to be responsible for methanotrophic activity in dioxygen-limited lakes (55). Finally, it has been speculated that alternatively or in conjunction, methanotrophs may form syntrophic partnerships with other microbes to facilitate methane oxidation (55) when dioxygen is limiting.

It should be noted, however, that in studies of methane oxidation in anoxic lake water samples, great care was taken to exclude any oxygen intrusion, and any trace amounts of oxygen present were quite small and could not explain the extent of methane oxidation observed. How these microbes then are able to oxidize methane in the absence of dioxygen is still unclear. That is, for the identified methanotrophs to oxidize methane, dioxygen is required for either form of MMO regardless of whether the alternative terminal electron acceptors can be used or an effective microbial partnership(s) can be formed. Thus, either unknown sources of dioxygen exist in these environments or these microbes possess some novel, as-yet-undescribed mechanism of anaerobic methane oxidation, i.e., novel forms of MMO that can utilize oxidized sulfur and nitrogen species in place of dioxygen.

Here, we present an alternative explanation for the presence and activity of aerobic methanotrophs in anoxic environments, particularly alphaproteobacterial methanotrophs. That is, genes for MB biosynthesis have been found only in the genomes of various *Methylosinus* and *Methylocystis* species of the *Alphaproteobacteria* (31). It has been repeatedly shown that these genera prefer high-methane/low-oxygen conditions found at the oxic-anoxic interface *in situ* (57, 58). Furthermore, they are the predominant methanotrophic genera present in completely anoxic zones of rice paddy soils (59). Thus, it is tempting to speculate that the ability to produce MB enables methanotrophs to colonize methane-rich environments by self-producing dioxygen to ensure that methane oxidation can continue even when ambient concentrations of dioxygen are quite low. Such a strategy is particularly important for methanotrophs that colonize the oxic-anoxic interface in soils because not only are these locations dark (thus excluding the possibility of methanotrophy/phototrophy synergy), this interface also shifts quickly in response to episodic precipitation and drying periods. As such, methanotrophs that colonize this interface must be prepared to tolerate periodic and possibly quite extended anoxic conditions. The ability to produce dioxygen from water would thus enable these microbes to continue to oxidize methane under anoxia, thereby generating ATP, as well as providing intermediates required for carbon assimilation (i.e., formaldehyde). Doing so would enable them to survive extended periods in the absence of oxygen, if not allowing for some continued growth under anoxic conditions.

It should be noted, however, that in the above-mentioned lake studies concluding that aerobic methanotrophy occurs in anoxic environments, gammaproteobacterial methanotrophs appeared to be predominantly responsible for methane oxidation, and to date, no representatives of this group have been shown to have the genes required for MB biosynthesis, although it is clear that at least some can and do secrete a copper-binding compound (60). It may be that these methanotrophs utilize dioxygen created by others via MB production (i.e., some sort of collaboration between gamma- and alphaproteobacteria as concluded between methanotrophs and oxygenic photosynthetic microbes) and/or can generate dioxygen via some unknown mechanism.

Finally, prior to the discovery of dioxygen production via the splitting of water by metal-MB complexes reported here, dioxygen production by biological systems has been observed in only four known pathways: oxygenic photosynthesis (61, 62), detoxification of oxygen radicals (63, 64), (per)chlorate respiration (65), and nitric oxide dismutation by “*Candidatus* Methylomirabilis oxyferans” of the NC10 phylum (66). The latter two mechanisms may provide some explanation for the significance of MB-mediated water oxidation. That is, it has been shown that dioxygen evolution from (per)chlorate respiration occurs when the intermediate chlorite is dismutated to chloride and dioxygen, and it is speculated that the dioxygen is then used for an antibiotic-producing monooxygenase in Haloferax volcanii (67). Furthermore, “*Ca*. Methylomirabilis oxyferans” is a methanotroph but respires nitrite rather than dioxygen. Interestingly, dioxygen is critical for its growth as this microbe utilizes the membrane-associated methane monooxygenase for methane oxidation to methanol (66). Stable-isotope studies showed that “*Ca*. Methylomirabilis oxyferans” dismutates nitric oxide to dinitrogen and dioxygen, the latter of which is used for methane oxidation (the mechanism[s] by which this occurs, however, is still unknown). It may be that MB-expressing aerobic methanotrophs perform a similar feat to ensure that there is adequate dioxygen for continued MMO activity under hypoxic/anoxic conditions.

In conclusion, the discovery of water oxidation by specific metal-methanobactin complexes not only is unusual but also implies a strategy whereby aerobic methanotrophs can survive, if not thrive, under anoxic conditions. As such, MB-driven dioxygen generation may be an important but hitherto unrecognized process whereby methane emissions are regulated.

## MATERIALS AND METHODS

### Materials.

Anhydrous CuCl_2_ (Acros Organics, Geel, Belgium), HAuCl_4_ (Acros Organics, Geel, Belgium), HgCl_2_ (Acros Organics, Geel, Belgium), AgF (Acros Organics, Geel, Belgium), FeCl_3_ (Acros Organics, Geel, Belgium), NiCl_2_ (Acros Organics, Geel, Belgium), CoCl_2_ (Acros Organics, Geel, Belgium), NaMoO_4_ (Sigma-Aldrich), and KCl (Sigma-Aldrich) were stored in a desiccator under Ar_2_. H_2_^18^O was obtained from Cambridge Isotope Laboratories, Inc. (Andover, MA, USA), and ^18,18^O_2_ was obtained from Sigma-Aldrich. Ar_2_, ^16,16^O_2_, and chemically pure (CP)-grade CH_4_ were obtained from Airgas USA LLC. High-performance liquid chromatography (HPLC)-grade acetonitrile, methanol, and other reagents/chemicals were purchased from Fisher Scientific and used without additional purification. Dianion HP-20 was purchased from Sigma-Aldrich LLC.

### Organisms, culture conditions, and isolation of methanobactin.

*Methylocystis* strain SB2 and *M. trichosporium* OB3b were cultured in nitrate mineral salts (NMS) medium (68) amended with either 0.2 or 1.0 μM CuSO_4_ to optimize the production of their methanobactin (MB-SB2). MB-SB2 was purified from the spent medium as previously described (69), with the following exception. The freeze-dried sample from the Dianion HP20 column was resuspended in deionized H_2_O and loaded onto a 250-mm by 20-mm Targa C_18_ column (Higgins Analytical, Inc., Mountain View, CA, USA) on an Azura HPLC system (Knauer, Berlin, Germany). MB-SB2 eluted in the 12 to 25% methanol fraction in a methanol-H_2_O gradient. The purified methanobactin was then freeze-dried as described above.

### X-ray photoelectric spectroscopy.

X-ray photoelectric spectroscopy (XPS) was performed as previously described (38, 43), with the following modifications. Samples containing HAuCl_4_ and HAuCl_4_ plus MB-SB2 were dried onto highly oriented pyrolytic graphite by freeze-drying. The 1-cm^2^ graphite substrates were immersed in a solution containing either HAuCl_4_ or HAuCl_4_ plus MB-SB2, frozen in liquid nitrogen, and lyophilized overnight. The graphite was then mounted onto an XPS puck and analyzed. Other drying methods were employed, such as drying in air under a stream of He gas with a drying time of 30 min or filtering through a porous alumina filter followed by a 2-min drying time. However, samples produced by these methods showed additional reduction.

As previously observed (38), XPS analysis of Au was complicated by X-ray-induced reduction during the measurement process. Au 4f peak areas were therefore measured as a function of X-ray exposure, the peak areas for a given X-ray dose were determined using the CASA XPS fitting program, and the areas were plotted as a function of time. An exponential fit to the data using the Igor Pro fitting program allowed the determination of the unirradiated sample’s Au^3+^ and Au^0^ peak areas.

XPS measurements were carried out on a custom-designed system that incorporated a Specs hemispherical analyzer (Specs Scientific Instruments, Sarasota, FL, USA), an Al X-ray source, and a load lock to allow rapid sample exchanges.

### Kinetics of Au^3+^ binding.

The rates of Au^3+^ binding to MB-SB2 were determined by measuring the absorption changes at 338 nm and 387 nm using a four-syringe Biologic SFM/4000/S stopped-flow reactor coupled to a MOS-500 spectrophotometer (Biologic Science Instrument SA, Claix, France) at 4°C as previously described (39). In contrast to the absorbance maxima using a Cary 50 spectrometer, the absorbance maximum for the oxazolone was 338 nm and that for the imidazolone ring was 387 nm with this system. Stock solutions of HAuCl_4_ were prepared in >18 MΩ · cm H_2_O. The stock solutions for MB-SB2 were prepared by dissolving freeze-dried MB-SB2 in >18 MΩ · cm H_2_O. Final concentrations of the stock solutions of MB-SB2 were determined after filtration by UV-visible absorption spectroscopy as previously described (39). The path length for the cuvette used in the Biologic SFM/4000/S stopped-flow reactor was 1.5 mm, and the dead time of the system was 1.4 ms. The system was cooled and maintained at 4°C. Reaction mixtures contained 400 μM MB-SB2 and either 40, 100, 200, 240, 280, 320, 360, 400, 600, 700, or 800 μM HAuCl_4_. Rates obtained for each concentration were averages from either 5 or 7 traces. The rates were determined by fitting the traces to the exponential function in Biokine operational software (Biologic Science Instrument SA). Binding rates were calculated in moles of Au bound per second per mole of MB-SB2 and are reported as the binding number (per second).

Fluorescence changes over time were monitored at 429 nm on a Cary Eclipse instrument (Agilent Technologies, Inc., Santa Clara, CA, USA) following excitation at 341 nm.

### Water oxidation.

Saturated solutions of anhydrous CuCl_2_, HAuCl_4_, HgCl_2_, AgF, FeCl_3_, NiCl_2_, CoCl_2_, NaMoO_4_, and KCl were prepared in a Coy anaerobic chamber (atmosphere of 95% Ar and 5% H_2_) (Coy Laboratory Products, Ann Arbor, MI, USA). The oxidation of 2H_2_O to O_2_ plus 4H^+^ in reaction mixtures containing a metal ion and either MB-SB2 or MB-OB3b was determined by monitoring the production of ^18,18^O_2_, H^+^, and, in the case of HAuCl_4_, Cl^−^. In oxygen evolution experiments, freeze-dried MB-SB2, MB-OB3b, and catalase as well as an anhydrous metal stock solution were prepared in 97% H_2_^18^O (Sigma-Aldrich, St. Louis, MO, USA) in 0.8-ml brown airtight vials (DWK Life Sciences, Millville, NJ, USA). Reaction mixtures contained 0 or 2 mM MB-SB2 or MB-OB3b and 0 to 20 mM metal ion in a final volume of 100 μl H_2_^18^O. Reaction mixtures were prepared in 2-ml brown serum vials sealed with Teflon-lined silicone septa. Initial experiments were performed with aluminum foil-wrapped vials, but that practice was discontinued once it was clear that identical results were produced regardless of whether the vials were wrapped or not. The generation of ^18,18^O_2_ from H_2_^18^O was monitored by the direct injection (1 μl or 2 μl) of headspace.

Gas samples were manually injected into an Agilent (Santa Clara, CA, USA) 7890B gas chromatography (GC) system with a 7250 accurate-mass quadrupole time of flight (Q-TOF) GC-mass spectrometry (MS) system and a DB5-ms column. Except for the ^18,18^O_2_ injections for standard curves, all injection volumes were 1 μl using gastight Hamilton syringes. Standard curves were generated with 1-μl, 1.5-μl, and 2-μl injections of 97% ^18,18^O_2_ (Sigma-Aldrich, St. Louis, MO, USA). The headspace in the vials was sampled before and after the addition of the metals, as was the outside air in the mass spectroscopy instrument, as controls. After the standards and controls were injected, the samples were mixed, and headspace samples were immediately collected, with subsequent samples being taken every 30 to 60 s. After several minutes, collection slowed to 1 sample every 2 to 3 min. The quantization of generated ^18,18^O_2_ came from an extracted-ion chromatogram set to 35.9978 Da. A small shift in the MS location of the ^18,18^O_2_ was observed on some dates. If a drift in the MS location of ^18,18^O_2_ was observed, the identity of the peak was verified with the 97% ^18,18^O_2_ standard.

### Oxidase, superoxide dismutase, hydrogen peroxide reductase, and iron reductase activities and pH measurements.

Oxidase, superoxide dismutase, and hydrogen peroxide reductase activities were determined as previously described by Choi et al. (48). A ferrozine assay was used to determine iron reductase activity (50, 51).

pH changes during metal titrations were monitored on either a PHM 220 pH meter with a pH2005-7 combined pH electrode (Radiometer Analytical, Villeurbanne, France) or an Oakton Ion 700 pH meter (Cole-Parmer, Vernon Hills, IL, USA).

Free chloride produced from the binding and deduction of HAuCl_4_ to Au^0^ was measured via argentometric titration (70). HAuCl_4_–MB-SB2 solutions were prepared at a molar ratio of 9:1 and incubated for at least 72 h. Following the incubation period, the solution was titrated with a standardized AgNO_3_ solution, delivered with a ramé-hart 2.0-ml microsyringe. The titration processes were monitored with a custom-made Ag wire working electrode and an Ag/AgCl reference electrode.

### Methane oxidation coupled to O_2_ generated from Cu^2+^ reduction by MB-OB3b. (i) Sample preparation.

*M. trichosporium* OB3b was grown on NMS medium (68) at 30°C in a 250-ml sidearm flask sealed with rubber stoppers. Cultures were shaken at 200 rpm under a methane-to-air ratio of 1:2 until the mid-exponential phase (optical density at 600 nm [OD_600_] of ∼0.3) was reached. Two milliliters of the cell culture was then transferred to 8.5-ml serum vials containing a Teflon-coated magnetic stir bar. Four separate conditions were used: (i) *M. trichosporium* OB3b with no amendments, (ii) *M. trichosporium* OB3b plus 25 μM copper (5 μl added from a filter-sterilized [0.22-μm] 10 mM stock solution of CuCl_2_), (iii) *M. trichosporium* OB3b plus 5 μM MB-OB3b (10 μl added from a filter-sterilized [0.22-μm] 1 mM stock solution of MB-OB3b), and (iv) *M. trichosporium* OB3b plus 25 μM copper and 5 μM MB-OB3b. Biological triplicate samples were prepared for all conditions. The vials were then crimp sealed and degassed using prepurified-grade filter-sterilized (0.22-μm) nitrogen gas (N_2_ [99.998%]) for 20 min at a flow rate of 3.42 ml/s using 22- and 25-gauge needles for N_2_ gas flow in and out. After degassing, the needles were removed, and the samples were immediately placed in an anaerobic chamber filled with an H_2_-N_2_ gas mixture (1:9 mixing ratio). Once in the anaerobic chamber, 1 ml of ^13^C-labeled methane (^13^CH_4_ [99%]) (Sigma-Aldrich, St. Louis, MO) was added using a 10-ml gastight syringe (Hamilton Company, Reno, NV). Vacuum grease was then spread on the top of the sealed septa. The vials were finally covered with aluminum foil and incubated inverted (septum side down) on a magnetic stir plate in the anaerobic chamber for 3 days at 25°C.

### (ii) Gas chromatography-mass spectrometry analysis.

GC-MS analyses were performed using an Agilent 7890B gas chromatograph system coupled with an Agilent 5977B single-quadrupole mass spectrometer (Agilent Technologies, Santa Clara, CA, USA). A Carboxen-1010 Plot capillary column (30 m by 0.32 mm) was used for separation (Supelco, Bellefonte, PA). Ten microliters of the headspace gas of each sample was injected manually using a 25-μl gastight syringe (Hamilton Company, Reno, NV). GC system conditions were as follows: He as the carrier gas at a flow rate of 10 ml/min, split injection with a split ratio of 5:1, an inlet temperature of 170°C, and an oven temperature maintained at 145°C throughout the analysis. The mass spectrometry ion source and quadrupole temperatures were 250°C and 200°C, respectively. Under these conditions, ^13^CH_4_ and ^13^CO_2_ were detected at 2.16 min and 2.86 min, respectively. Data were acquired in selected ion monitoring (SIM) mode, monitoring *m/z* 17 for ^13^CH_4_ and *m/z* 45 for ^13^CO_2_.
