# Supplementary material for: Oxygen Generation via Water Splitting by a Novel Biogenic Metal Ion-Binding Compound
Source: Appl Environ Microbiol. 2021 Jun 25;87(14):e00286-21. doi: 10.1128/AEM.00286-21 (PMC8231713; doi:10.1128/AEM.00286-21)
Supplement: Supplemental file 1 — supplemental text, Fig. S1 to S6, Tables S1 to S3. Download AEM00286-21_Supp_1_seq2.pdf, PDF file, 3.5 MB [file aem00286-21_supp_1_seq2.pdf]

Revised Manuscript: AEM00286-21

Supplemental Information

Oxygen generation via water splitting by a novel biogenic metal ion binding compound

Philip Dershwitz[1], Nathan L. Bandow[1Ψ], Junwon Yang[2], Jeremy D. Semrau[2], Marcus T. McEllistrem[3], Rafael A. Heinze[3], Matheus Fonseca[3], Joshua C. Ledesma[1], Jacob R. Jennett[1], Ana M. DiSpirito[1], Navjot S. Athwal[1ϒ], Mark S. Hargrove[1], Thomas A. Bobik[1], Hans Zischka[4], and Alan A. DiSpirito[1]*

[1]Roy J. Carver Department of Biochemistry, Biophysics and Molecular Biology. Iowa State University, Ames, IA 50010-3260, USA

[2]Department of Civil and Environmental Engineering, University of Michigan, Ann Arbor, MI, 48109-2125, USA

[3]Department of Chemistry, University of Wisconsin-Eau Claire, WI, 54602, USA

[4] Institute of Molecular Toxicology and Pharmacology, Helmholtz Center Munich, German Research Center for Environmental Health, Ingolstaedter Landstrasse 1, D-85764 Neuherberg, Germany. and Technical University Munich, School of Medicine, Institute of Toxicology and Environmental Hygiene, Biedersteiner Strasse 29, D-80802 Munich, Germany.

[Ψ] Current address: Allogene Therapeutics, Inc., South San Francisco, CA 94080, USA

[ϒ]Current address: Stemcell Technologies, Vancouver, BC Canada

MATERIALS AND METHODS

UV-visible absorption, fluorescence and circular dichroism

UV-visible absorption spectroscopy was performed using either a Cary 50 (Agilent Technologies Inc. Santa Clara, CA, USA) or using an Ollis Cary 14 (Ollis Inst. Sys. Bogart, GA, USA) spectrophotometer. Fluorescence spectroscopy scans and titrations were performed on a on a Cary Eclipse (Agilent Technologies Inc. Santa Clara, CA, USA). Reaction mixtures contained either 40 or 50µM MB-SB2 and were titrated with a 10mM $HAuCl_4$ solution. Time course spectra were performed with 50 µM MB-SB2 solutions containing 112.5 µM $HAuCl_4$. Scans were taken every 5 min for 1 h or every 60 min for 48 h.

For excitation fluorescence maxima, spectra were taken at a scan rate of 30 nm sec^{-1} between 290 and 700 nm with excitation and emission slits at 5 nm and a photomultiplier tube voltage of 600 V. Excitation wavelengths of 285, 341 or 394nm were used.

Circular dichroism (CD) spectra were recorded on a JASCO-J-710 spectropolarimeter (Jasco Co. Tokyo, Japan). Solution preparations and titrations were determined as described above for fluorescence measurements.

Isothermal Titration Calorimetry (ITC)

Isothermal titration calorimetry (ITC) was performed at 25°C using a GE Microcal ITC200 microcalorimeter (GE Healthcare, Piscataway, NJ, USA). The stock titration solution was 2 mM $HAuCl_4$, and the cell contained 100 µM MB-SB2. Injections were added at 180 sec intervals, and injection volumes varied from 1 – 2 µl with a stir rate of 1000 rpm. The instrument was cleaned between experiments according to the manufacturer's recommendation followed by a rinse

with 100µM MB-SB2 to remove residual metal. Data were analyzed using nonlinear least-squares curve fitting in Origin 7.0 software (GE Healthcare, Piscataway, NJ, USA) or by CHASM software (1) following subtraction of the heat of dilution of $HAuCl_4$ into H_2O. Due to fitting limitations of the Origin software some of the data were fit manually. Manual fits were performed until the Origin software's fitting algorithm recognized the fit. After the Origin software recognized the fit, the fitting algorithm was used to minimize the χ^2 values. Due to the complexity of the titration curve, the data were divided into sections and each section modeled separately when using Origin software. The Origin software requires the data to return to zero in order to be fit. In the case of the initial binding event the entire curve was shifted along the y-axis by 53,600 calories per mole to allow the curve to be fit. ΔH values where then corrected by the magnitude of the shift before calculating ΔG. All four assumed binding constants could be fit simultaneously using the CHASM software.

Nanoparticle formation and transmission electron microscopy (TEM)

Gold nanoparticles were prepared in reaction mixtures containing 25 µM MB-SB2 plus 56.25 µM $HAuCl_4$, 50 µM MB-SB2 plus 45 µM $HAuCl_4$, 50 µM MB-SB2 plus 112.5 µM $HAuCl_4$, 50 µM MB-SB2 plus 225 µM $HAuCl_4$, or 50 µM MB-SB2 plus 500 µM $HAuCl_4$. Samples were incubated at 25°C for 10 to 51,840 min (36 days) with and without stirring. Samples were spotted on formvar-coated 200 mesh nickel grids. Transmission electron microscopy was performed on a JEOL 2100 200kV scanning and transmission electron microscope (Japan Electron Optics Laboratory, Peadbody, MA, USA). Nanoparticles were counted and sized using Anally software (Japan Electron Optics Laboratory).

RESULTS

Circular Dichroism

In contrast to the fluorescent spectra, no exciton coupling was observed in the visible CD spectra between the oxazolone and imidazolone rings in MB-SB2 (Fig. S3). The blue shift, weak signal and absence of an expected Cotton effect suggested the two chromophores were in a parallel orientation in the as isolated MB-SB2 sample (2, 3). At molar ratios of $HAuCl_4$ to MB-SB2 ratios between 0.2 and 0.9, exciton coupling was observed with maximal exciton coupling amplitude at $HAuCl_4$ to MB-SB2 ratio of 0.5, suggesting the rings are in an oblique orientation in the dimer stage. At equimolar concentrations of $HAuCl_4$, exciton coupling was lost and weak positive signals for both the oxazolone and imidazolone groups were observed at 353 and 414nm, respectively. In addition, the decreased signal at 210 nm, at 1.0 $HAuCl_4$ per MB-SB2 from both the oxazolone and imidazolone groups suggest a structured molecule where the rings are in an in-line orientation.

Isothermal Titration Calorimetry (ITC)

Four separate trends were observed in ITC experiments titrating $HAuCl_4$ to MB-SB2 (Fig. S4; Table S1). As a result of this complexity, the fitting using Origin software was divided into three different sections and a two-site model was used for each section. Further, due to the unusual shape of the initial binding trend as well as the limitations of the Origin software, the entire curve was shifted by 53,600 cal mol^{-1} in order to fit the data. The ΔH values were then corrected. Following this adjustment, the initial binding constant was estimated to be 1.97 x

$10^{34} \pm 1.7 \times 10^{13}\ M^{-1}$ (Table S1). This initial binding constant was greater than the initial binding constant for Cu^{2+} (4)

The thermodynamic changes following the addition of $HAuCl_4$ to MB-SB2 was also analyzed by using the multiple-site binding model software, CHASM (Figure S4D). The calculated thermodynamic data for both Origin and CHASM were similar (Table S1). It should also be noted that initial binding constants listed in Table S1 are beyond the working range of the ITC system from Microcal and can only be considered estimated values, although the displacement of Cu^{+} from Cu-MB-SB2 by $HAuCl_4$ suggest the binding constant is > $10^{25}\ M^{-1}$.

Nanoparticle Formation

Although not initially observed in UV-visible absorption spectra following addition of high (>1.5) molar ratios of $HAuCl_4$ to MB-SB2, a color change from yellow to red-purple was observed over time. Subsequently, this color change was monitored over time in reaction mixtures containing 2.25 $HAuCl_4$ per MB-SB2. The results showed the development of a peak at 527 nm, which increased throughout the 50h incubation period (Figure S5). The absorption maximum at 527 nm is characteristic of small (< 10 nm) spherical gold nanoparticle (5). Gold nanoparticles were first observed after approximately 10 min (Figure S5B).

Nanoparticle size and shape was stable in solution for up to 36 days at the $HAuCl_4$ to MB-SB2 ratio of 2.25 with an approximate diameter of 2.0nm (Figure S5D; Table S2). However, after approximately 6 hours, nanoparticles began to group into clusters and eventually aligned into string-like structures (Fig. 5D). This clustering may be responsible for the color change and

the increased emission at 527 nm as the optical properties of nanoparticles have been shown to be affected by distance between nanoparticles (5).

DISCUSSION

As observed with the methanobactin from *Methylosinus trichosporium* OB3b, the spectral and thermodynamic properties of MB-SB2 suggest this peptide coordinates Au^0 in different oligomeric forms depending on the $HAuCl_4$ to MB-SB2 ratio with the highest binding constant observed at low (< 0.25) $HAuCl_4$ to mb-SB2 ratios. The formation of both monomeric and oligomeric metal coordination appears similar to the to that observed in synthetic heterocyclic thioamides (5). The clustering of Au-MB-SB2 over time into polymer-like structures is also similar to that observed in some metal heterocyclic thioamide complexes (5). In addition to the number of Au^{3+} reduced to Au^0, gold nanoparticle formation by MB-SB2 was unique in the consistency of particle size and stability in aqueous solution.

FIGURE LEGENDS

Figure S1. UV-visible absorption spectra of 40µM SB2-MB as isolated and following thirty three 0.0125 molar additions of $HAuCl_4$ (**A**), followed by eight 0.125 molar additions of $HAuCl_4$ (**B**) and followed by another eleven 0.125 molar additions of $HAuCl_4$ (**C**)**. (D)** Absorbance of the oxazolone (○) and imidazolone (△) groups at 336 and 387 nm, respectively as a function of $HAuCl_4$ to MB-SB2 molar ratios. Initial MB-SB2 and $HAuCl_4$ solutions were identical to the solutions used in the pH titration in figure 1. Abbreviations: Oxa, oxazolone; Imi, imidazolone.

Figure S2. Emission spectra from MB-SB2 following excitation at 285nm (**A**), 341nm (**B**) and 394nm (**C**) as isolated (—) and following the addition of 0.25 (—), 0.5 (—), 1.0 (—), 1.5 (—) and 2.0 (—) $HAuCl_4$ per MB-SB2. **D**, Emission at 419nm (○) and at 456nm (△) following excitation at 341 and 394 nm, respectively, and following 0.05 molar additions of $HAuCl_4$.

Figure S3. **A.** UV-visible CD spectra of MB-SB2 as isolated (—) and following the addition of 0.5 (—), 1.0 (—) and 2.25 (—) $HAuCl_4$ per MB-SB2. **B.** Molar ellipticity changes at 210 (◇), 336 (○) and at 378 (△) nm following additions of $HAuCl_4$. **Insert.** Molar ellipticity changes at 353 (●) and at 414 (△) nm following additions of $HAuCl_4$. Abbreviations: Oxa, oxazolone; Imi, imidazolone.

Figure S4. **A-C**. Binding isotherms from Origin software of $HAuCl_4$ titrations to MB-SB2 and following subtraction of the heat of dilution of $HAuCl_4$ into H_2O. Due to the complexity of the titration and limitations of the Origin software the data was

divided to allow fitting. **D**. Screen capture of the MATLAB user interface of CHASM software showing a four-binding-site fit of the ITC data shown in panels A - C.

Figure S5. Spectral changes and nanoparticle formation overtime by MB-SB2 following the addition of 2.25 $HAuCl_4$ per MB-SB2. (**A**) UV-visible spectra of taken every 5 min following the addition of $HAuCl_4$ to MB-SB2. (**B**) Absorption changes at 336 (○), 387 (△) and 537 (○) nm, insert TEM of nanoparticles formed by MB-SB2 10 min after the addition of $HAuCl_4$. (**C**) Absorption changes at 537 (○) nm, insert TEM of nanoparticles formed by MB-SB2 6, 24 and 59 hours after the addition of 2.25 $HAuCl_4$. (**D**) TEM of nanoparticles formed after 36 days in a solution containing 2.25 $HAuCl_4$ per MB-SB2.

Fig. S6. **(A)** UV-visible absorption spectra of 40µM SB2-MB as isolated and following molar additions of KCl (A), $NaMoO_4$ (B) AgF (C), $HgCl_2$ (D), $FeCl_3$ (E), $NiCl_2$ (F), or $CoCl_2$ (G). Panel D insert, equimolar $FeCl_3$ MB-SB2 solution.

Table S1.

Thermodynamic parameters as calculated by Origin and by CHASM for $HAuCl_4$ binding to MB-SB2.

Parameter	Tetramer	Dimer	Monomer	Nanoparticle Formation
$HAuCl_3$ Origin				
T_K	298.15	298.15	298.15	298.15
N (Au:mb-SB2^{-1})	0.207	0.447	1.10	0.380
***K* (M^{-1})**	**$1.97 \times 10^{34} \pm 1.73 \times 10^{13}$**	**3.65×10^{8}**	**7.87×10^{11}**	**3.09×10^{10}**
ΔH (cal • mol^{-1})	-71,560*	-93,900	-68,190	-5,689
ΔS (cal^{-1} • mol^{-1} • deg^{-1})	96.7	-276	-174	28.9
ΔG (cal•mol^{-1})	-100,391	-11,611	-16,312	-14,306
**χ^2**	2.40×10^{9}	7.25×10^{6}	5.97×10^{5}	5.97×10^{5}
$HAuCl_3$ CHASM				
N (Au:mb-SB2^{-1})	0.21 ± 5.83	0.67 ± 2.18	0.36 ± 4.33	0.24 ± 2.20
***K* (M^{-1})**	**$4.97 \times 10^{35} \pm 6.63 \times 10^{24}$**	**$3.54 \times 10^{8} \pm 4.8 \times 10^{8}$**	**$1.60 \times 10^{7} \pm 2.23 \times 10^{7}$**	**$2.35 \times 10^{10} \pm 3.55 \times 10^{10}$**
ΔH (cal • mol^{-1})	-78,140 ± 5,800	-62,050 ± 2,200	-9,841 ± 4,300	-91,490 ± 2,200
ΔS (cal^{-1} • mol^{-1} • deg^{-1})	-29.47	-50.4	0.02	77.35
ΔG (cal•mol^{-1})	-48,700	-11,700	-9,820	-14,100

*calculated value following data shift

Table S2

Nanoparticle size distribution in reaction mixtures containing 2.25 $HAuCl_4$ per MB-SB2.

**MB-SB2 µM**	**Time (h)**	**Average Diameter (nm)**	**Percent X ≤ 3 (nm)**	**3 > X ≤ 10 (nm)**	**10 > X ≤ 15 (nm)**	***n***
25	6	1.9 ± 0.5	99.1	0.8	0.1	2565
25	24	2.0 ± 0.3	99.0	0.9	0.1	258
50	6	1.8 ± 0.4	99.3	0.7	0	715
50	24	2.0 ± 0.4	98.2	1.8	0	1099
100	6	2.0 ± 0.5	99.1	0.9	0	683
250	6	2.0 ± 0.4	97.1	0.9	0	280

Table S3

Superoxide dismutase (SOD), oxidase and hydrogen peroxide reductase (HPR) activities of MB-SB2, copper containing MB-SB2 (Cu-MB-SB2) and gold containing MB-SB2 (Au-MB-SB2). NADH (0.5 mM) was used as the reductant for oxidase and HPR activities.

**MB**	**SOD**	**Oxidase**	**HPR**
	**($O_2^{\bullet -}$ min^{-1} mb^{-1})**	**(O_2 min^{-1} mb^{-1})**	**(H_2O_2 min^{-1} mb^{-1})**
MB-SB2*	-	0.5 ± 0.01	-
Cu-mb-SB2*	$2{,}350 \pm 420$	0.9 ± 0.05	18 ± 0.02
Au-MB-SB2	$1{,}980 \pm 37$	0.7 ± 0.01	11 ± 0.03

*From (6)

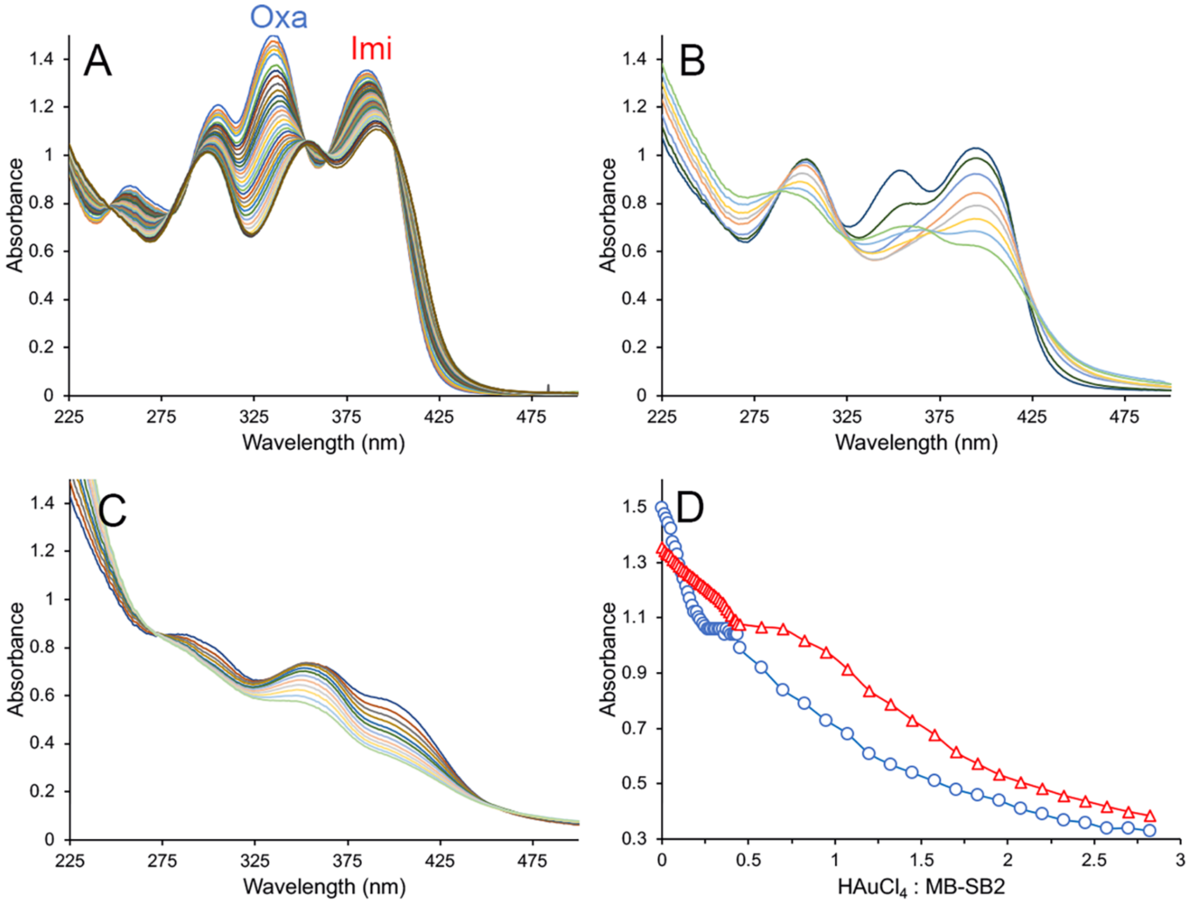

Figure S1. UV-visible absorption spectra of 40µM SB2-MB as isolated and following thirty three 0.0125 molar additions of $HAuCl_4$ (**A**), followed by eight 0.125 molar additions of $HAuCl_4$ (**B**) and followed by another eleven 0.125 molar additions of $HAuCl_4$ (**C**). **D.** Absorbance of the oxazolone (○) and imidazolone (△) groups at 336 and 387 nm, respectively as a function of $HAuCl_4$ to MB-SB2 molar ratios. Initial MB-SB2 and $HAuCl_4$ solutions were identical to the solutions used in the pH titration in Figure 1. Abbreviations: Oxa, oxazolone; Imi, imidazolone.

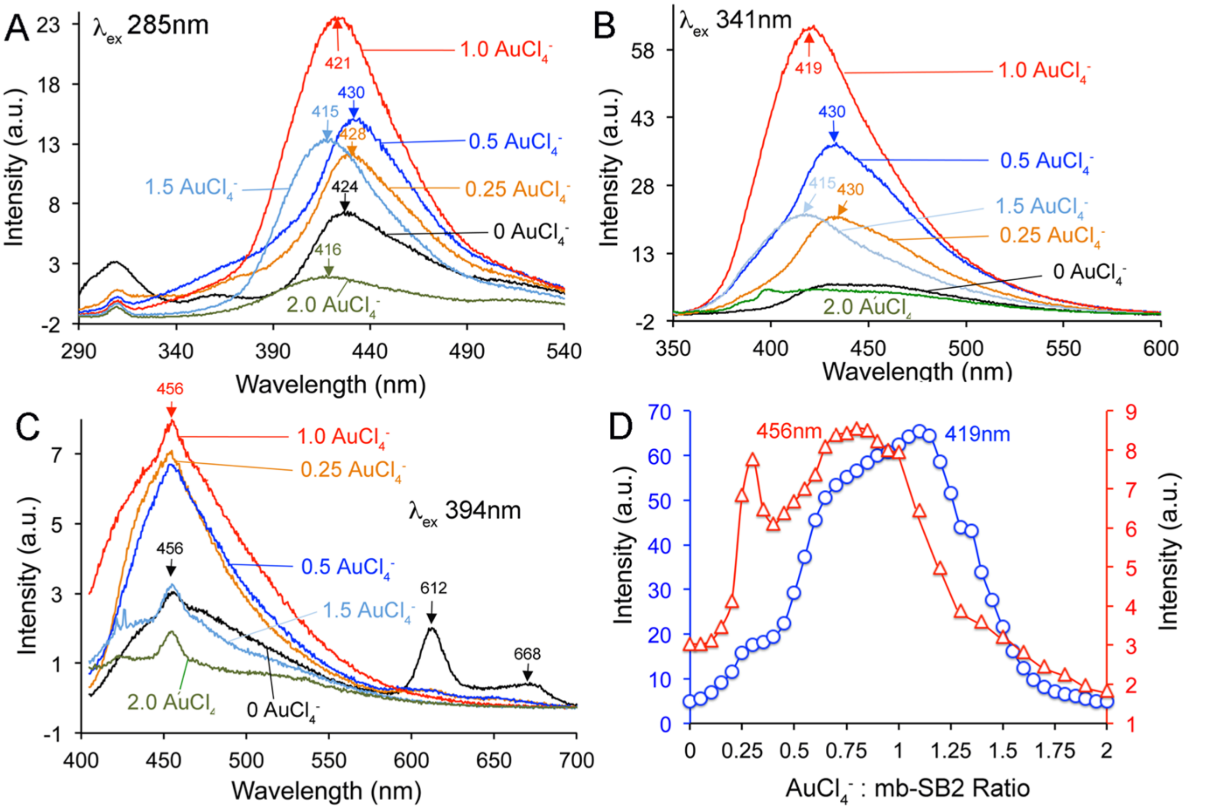

Figure S2. Emission spectra from SB2-MB following excitation at 285nm (**A**), 341nm (**B**) and 394nm (**C**) as isolated (—) and following the addition of 0.25 (—), 0.5 (—), 1.0 (—), 1.5 (—) and 2.0 (—) $HAuCl_4$ per MB-SB2. **D**, Emission at 419nm (○) and at 456nm (△) following excitation at 341 and 394 nm, respectively, and following 0.05 molar additions of $HAuCl_4$.

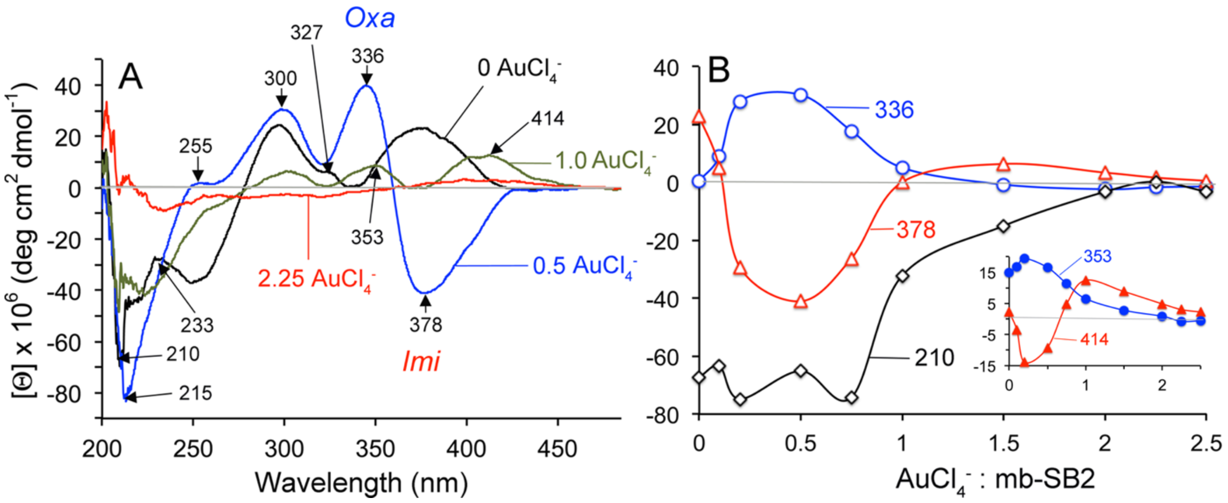

Figure S3. **A.** UV-visible CD spectra of MB-SB2 as isolated (—) and following the addition of 0.5 (—), 1.0 (—) and 2.25 (—) $HAuCl_4$ per MB-SB2. **B.** Molar ellipticity changes at 210 (◇), 336 (○) and at 378 (△) nm following additions of $HAuCl_4$. **Insert.** Molar ellipticity changes at 353 (●) and at 414 (△) nm following additions of $HAuCl_4$. Abbreviations: Oxa, oxazolone; Imi, imidazolone.

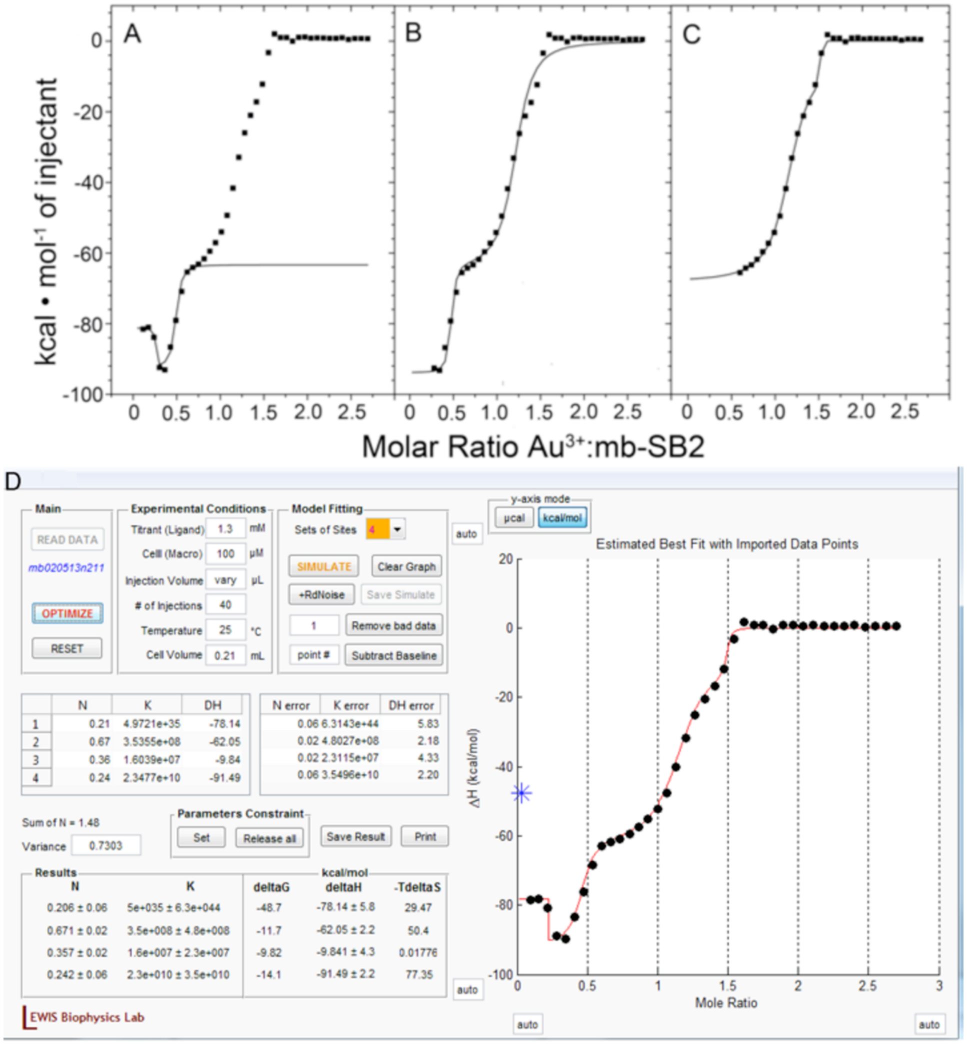

Figure S4. **A-C**. Binding isotherms from Origin software of $HAuCl_4$ titrations to MB-SB2 and following subtraction of the heat of dilution of $HAuCl_4$ into H_2O. Due to the complexity of the titration and limitations of the Origin software the data was divided to allow fitting. **D**. Screen capture of the MATLAB user interface of CHASM software showing a four-binding-site fit of the ITC data shown in panels A - C.

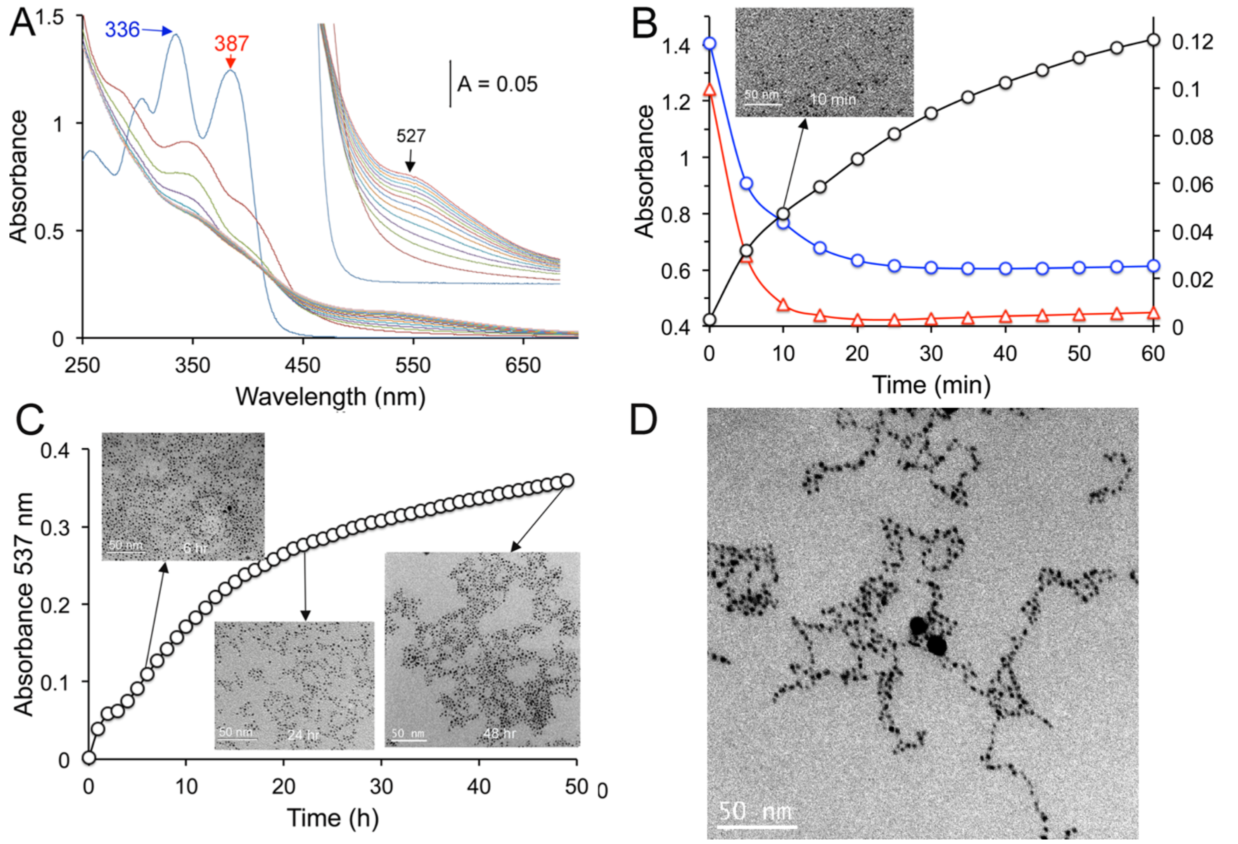

Figure S5. Spectral changes and nanoparticle formation overtime by MB-SB2 following the addition of 2.25 $HAuCl_4$ per MB-SB2. (**A**) UV-visible spectra of taken every 5 min following the addition of $HAuCl_4$ to MB-SB2. (**B**) Absorption changes at 336 (○), 387 (△) and 537 (○) nm, insert TEM of nanoparticles formed by MB-SB2 10 min after the addition of $HAuCl_4$. (**C**) Absorption changes at 537 (○) nm, insert TEM of nanoparticles formed by MB-SB2 6, 24 and 59 hours after the addition of 2.25 $HAuCl_4$. (D) TEM of nanoparticles formed after 36 days in a solution containing 2.25 $HAuCl_4$ per MB-SB2.

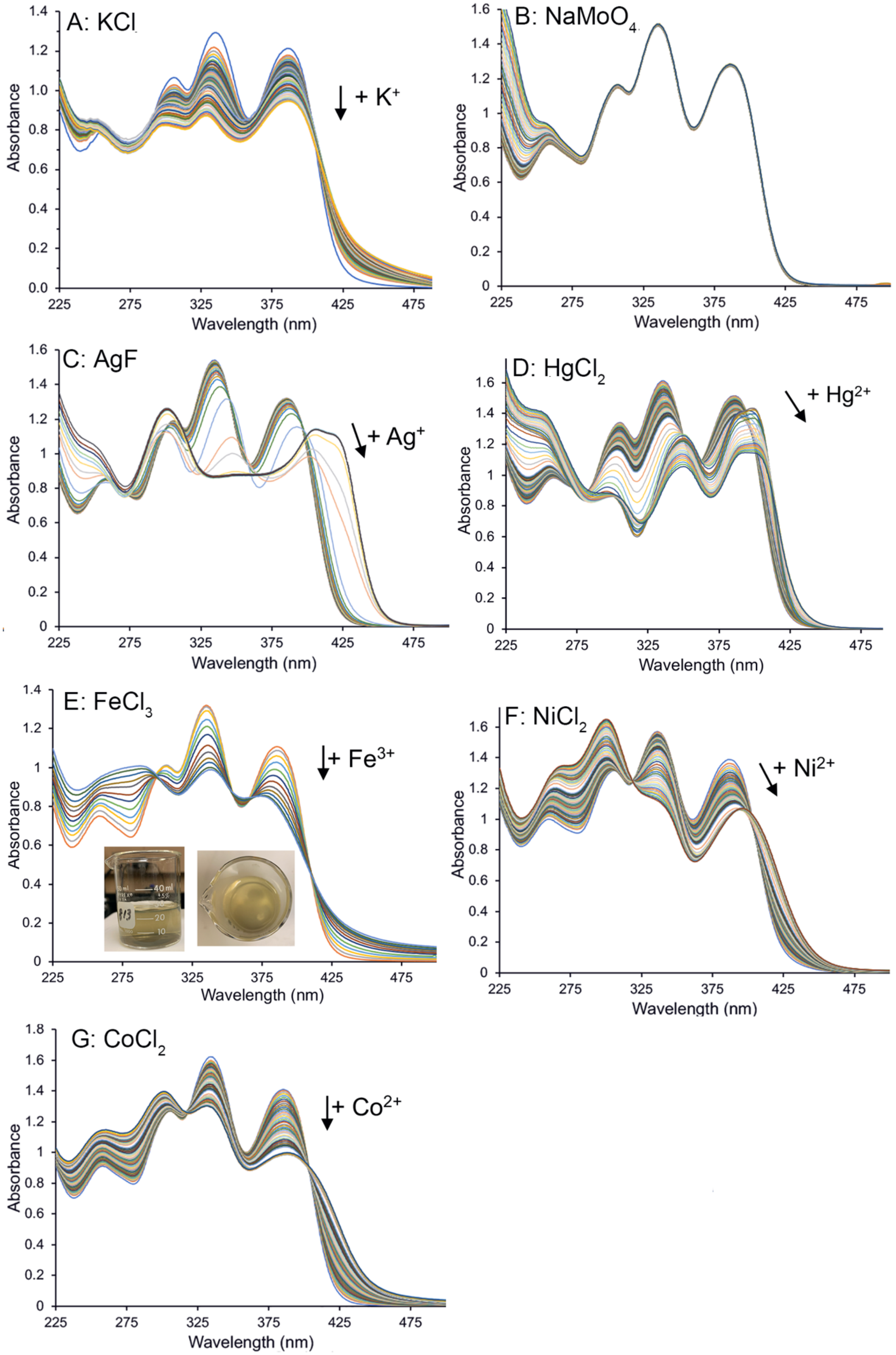
A: KCl
+ K+
B: NaMoO4
C: AgF
+ Ag+
D: HgCl2
+ Hg2+
E: FeCl3
+ Fe3+
F: NiCl2
+ Ni2+
G: CoCl2
+ Co2+
Absorbance
Wavelength (nm)

223

Fig. S6. (**A)** UV-visible absorption spectra of 40μM SB2-MB as isolated and following molar additions of KCl (A), $NaMoO_4$ (B) AgF (C), $HgCl_2$ (D), $FeCl_3$ (E), $NiCl_2$ (F), or $CoCl_2$ (G). Panel D insert, equimolar $FeCl_3$ MB-SB2 solution.

Supplemental Citations

1. Le VH, Buscaglia R, Chaires JB, Lewis EA. 2013. Modeling complex equilibria in isothermal titration calorimetry experiments: Thermodynamic parameters estimation for a three-binding-site model. Anal Biochem 434:233 - 241.
2. Berova N, Di Bari L, Pescitelli G. 2007. Application of electronic circular dichroism in configurational and conformational analysis of organic compounds. Chem Soc Rev 36:914 - 931.
3. Berova N, Nakagawa KH. 2000. Exciton chirality method: principles and applications, p 337 - 382. *In* Berova N, Nakagawa KH, Woody RW (ed), Circular Dichroism Principles and Applications, Second ed. Wiley_VCH, Inc, New York, NY USA.
4. Bandow N, Gilles VS, Freesmeier B, Semrau JD, Krentz B, Gallaghe W, McEllistrem MT, Hartse SC, Cho DW, Hargrove MS, Heard TM, Chesner LM, Braunreiter KM, Cao BV, Gavitt MM, Hoopes JZ, Johnson JM, Polster EM, Schoenick BD, A.M. U, DiSpirito AA. 2012. Spectral and copper binding properties of methanobactin from the facultative methanotroph *Methylocystis* strain SB2. J Inorgan Biochem 110:72 - 82.
5. Storhoff JJ, Lazarides AA, Mucic R, C.A. M, Letsinger RL, Schatz GC. 2000. What controls the optical properties of DNA-linked gold nanoparticle assemblies. J Am Chem Soc 122:4640-4650.

6. Choi DW, Semrau JD, Antholine WE, Hartsel SC, Anderson RC, Carey JN, Dreis AM, Kenseth EM, Renstrom JM, Scardino LL, Van Gorden GS, Volkert AA, Wingad AD, Yanzer PJ, McEllistrem MT, de la Mora AM, DiSpirito AA. 2008. Oxidase, superoxide dismutase, and hydrogen peroxide reductase activities of methanobactin from types I and II methanotrophs. J Inorg Biochem 102:1571-80.
